# The Morphological Changes in Adjacent Segments Amongst Patients Receiving Anterior and Oblique Lumbar Interbody Fusion: A Retrospective Study

**DOI:** 10.3390/jcm10235533

**Published:** 2021-11-26

**Authors:** Kuan-Kai Tung, Fang-Wei Hsu, Hsien-Che Ou, Kun-Hui Chen, Chien-Chou Pan, Wen-Xian Lu, Ning-Chien Chin, Cheng-Min Shih, Yun-Che Wu, Cheng-Hung Lee

**Affiliations:** 1 Department of Orthopedics, Taichung Veterans General Hospital, Taichung 40705, Taiwan; david02200918@gmail.com (K.-K.T.); orthochen@gmail.com (K.-H.C.); adonisvgh@gmail.com (C.-C.P.); orthobutterfly@gmail.com (N.-C.C.); 10chengmin@gmail.com (C.-M.S.); wcmin0806@gmail.com (Y.-C.W.); 2 Department of Orthopedics, Kuang Tien General Hospital, Taichung 433, Taiwan; fangwei0312@gmail.com; 3 Department of Medicine Education, Taipei Veterans General Hospital, Taipei 112201, Taiwan; knockthe2012@gmail.com; 4 Department of Biomedical Engineering, Hung Kuang University, Taichung 433304, Taiwan; 5 Department of Computer Science and Information Engineering, Providence University, Taichung 43301, Taiwan; 6 Department of Nursing, Jen-Teh Junior College of Medicine, Nursing and Management, Miaoli 35664, Taiwan; 7 Department of Rehabilitation Science, Jen-Teh Junior College of Medicine, Nursing and Management, Miaoli 35664, Taiwan; 8 Department of Orthopedics, Feng Yuan Hospital Ministry of Health and Welfare, Taichung 420, Taiwan; wslu.orth@gmail.com; 9 Department of Physical Therapy, Hung Kuang University, Taichung 433304, Taiwan; 10 PhD Degree Program of Biomedical Science and Engineering, College of Biological Science and Technology, National Yang Ming Chiao Tung University, Hsinchu 300093, Taiwan; 11 College of Medicine, National Chung Hsing University, Taichung 40227, Taiwan; 12 Department of Food Science and Technology, Hung Kuang University, Taichung 433304, Taiwan

**Keywords:** adjacent segment morphology, adjacent segment disease, ALIF, OLIF, minimal invasive surgery (MIS), short lumbar spinal fusion, radiographic outcome

## Abstract

Adjacent segment disease (ASD) is troublesome condition that has proved to be highly related to spinal malalignment after spinal surgery. Hence, we aimed to evaluate the morphological changes after anterior lumbar interbody fusion (ALIF) and oblique LIF (OLIF) to establish the differences between the two surgical methods in terms of possible ASD avoidance. Fifty patients, half of whom received ALIF while the other half received OLIF, were analyzed with image studies and functional outcomes during the pre-operative and post-operative periods, and 2 years after surgery. Image measurements obtained included spinal-pelvic parameters, index lordosis (IL), segmental lordosis (SL), anterior disc height (ADH), posterior disc height (PDH) and adjacent segment disc angle (ASDA). The ADH and PDH in the adjacent segment decreased in the two groups while OLIF showed greater decrease without radiological ASD noted at 2-year follow-up. Both groups showed an increase in IL after surgery while ALIF showed greater improvement. No statistical difference was identified in functional outcomes between LIFs. We suggest that both ALIF and OLIF can restore adequate lordosis and prevent ASD after surgery. However, it should be noted that patient selection remains crucial when making any decision involving which of the two methods to use.

## 1. Introduction

Spinal fusion after decompression remains the standard treatment for symptomatic low back pain in adults after the use of conservative strategies fails. Recently, the introduction and increased adoption of the lumbar interbody fusion (LIFs) technique, including anterior (ALIF) and oblique lumbar interbody fusion (OLIF), has been proven to be efficient in restoring lumbar lordosis (LL), index disc height, the central canal and foraminal area by inserting lordotic angular grafts in a minimally invasive manner [1].

On the contrary, surgical complications such as radiographic adjacent segment disease (ASD) have been reported with incidence rates ranging from 8% to 100%, and one-third of those cases considered as being symptomatic [1,2]. The term radiographic ASD is defined as radiographic changes in segments adjacent to the fusion levels occurring with or without clinical symptoms [3]. Additionally, symptomatic ASD may be diagnosed with clinical symptoms such as worsening back pain, leg pain, or even intermittent claudication during the post-operative (post-OP) follow-up. These problematic complications necessitate further surgical interventions and worsen the surgical outcomes [4]. Biomechanical changes, such as increasing segmental motion and mechanical stress are considered as precipitators of degenerative stress and ASD.

It has been proposed that the abnormal distribution of pressure resulting from reduced lordosis may lead to increased tension in the posterior column of adjacent segments [5]. Moreover, disability in patients with spinal deformities are strongly related to deviations in sagittal alignment [6]. Hence, the optimal strategies for preventing ASD may require adequate restoration of the spinal alignment and the contouring of interbody cages. As long as the implant cages of ALIF and OLIF have sufficient lordotic angles, restoring lordosis and the subsequent prevention of ASD is theoretically achievable.

However, a comparison between patients receiving ALIF and OLIF regarding morphological changes in the adjacent segment and spinal alignment has rarely been discussed. Thus, we aimed to evaluate morphological changes in the adjacent segments amongst patients receiving anterolateral LIFs as treatment for symptomatic lumbar disorders.

## 2. Materials and Methods

### 2.1. Study Subjects

Patients who had received lumbar interbody fusion, including ALIF and OLIF, for symptomatic alignment reconstruction in 2016 to 2018 were identified as study subjects at the Taichung Veterans General Hospital. All patients underwent both interbody fusion and posterior instrumentation. The ALIF and OLIF patients were matched by gender and then age-controlled for comparison. The selected fusion level must be less than level 4 since a level 5 correction may be considered as a deformity correction. The selection criteria for the patients were: (1) age > 20 years; (2) the presence of low back pain or sciatica caused by radiculopathy or spondylosis, which was unresponsive to conservative treatment for more than 6 months; and (3) the pre-operative (pre-OP), nearest post-OP outpatient department visit (post-OP), and 2-year follow-up clinical imaging data and follow-up records must be complete. The exclusion criteria included: (1) loss during follow-up; (2) spinal deformity due to the presence of an active infection, malignancy, trauma, or neuromuscular disease etiology; (3) patient had received previous lumbar surgery, including LIFs; (4) patient without full-length lateral spine radiographs at the pre-OP, post-OP, and 2-year follow-up time periods; and (5) hybrid LIF surgery, such as performing ALIF and OLIF simultaneously on the same patient. The “2-year follow-up period” was defined as the time from the nearest post-OP outpatient department visit to 2-year follow-up.

### 2.2. Operation Procedures

Two interbody fusion techniques were included in this study: ALIF and OLIF.

#### 2.2.1. ALIF Procedure

The ALIF was performed with the patient in the supine position. A longitudinal incision was performed to allow for appropriate spine level exposure. A blunt dissection for retroperitoneal exposure and the securing of the inferior epigastric, left common iliac vessels, and genitofemoral nerves was performed. The iliac vessels were then exposed and retracted laterally to reveal the fixation level. After X-ray positioning, the target annulus fibrosus was resected and intervertebral disc tissue was scraped. Curettage and serial distractors were used for efficient disc removal and disc height evaluation. A bone graft was prepared with an allograft or Actifuse. A cage implant (TM-400 or the Depuy Synthes Syncage system) was inserted under sufficient exposure. Position of the implant was confirmed through X-ray fluoroscopy. The wound was then closed after adequate hemostasis was performed.

#### 2.2.2. OLIF Procedure

The OLIF was performed with the patient positioned in the right lateral position. A longitudinal incision at the front line of the iliac crest was made for appropriate spine level exposure before the psoas muscle was gently retracted posteriorly. After X-ray positioning, the target annulus fibrosus was resected and intervertebral disc tissue scraped. Serial trial was applied for optimal cage size. Endplate damage was avoided during the procedure. A cage implant (Medtronic CLYDESDALE Spinal System) was inserted under sufficient exposure. Position of the implant was confirmed by X-ray fluoroscopy. The wound was then closed after adequate hemostasis was performed. At L5/S1 level, we greatly prefer ALIF over OLIF as it avoids the anatomical challenges of OLIF.

### 2.3. Radiographic Assessment

**Figure 1:**
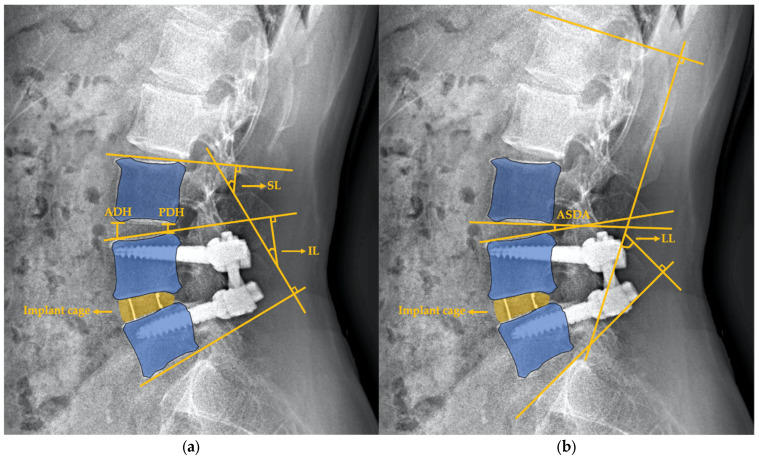
Schematic of radiographic measurement in morphological changes in adjacent segments. These figures showed L4/5 OLIF in situ. (**a**) Index lordosis (IL) and segmental lordosis (SL), anterior disc height (ADH), and posterior disc height (PDH) (**b**) Adjacent segment disc angle (ASDA) and lumbar lordosis (LL).

Full-length lateral spine radiographs for the kyphosis series (36 inch) were analyzed at three time points: pre-OP visit, post-OP visit and 2-year follow-up. All images were evaluated by 2 well-trained doctors (K.-K.T. and F.-W.H.) using validated Surgimap surgical planning software (Nemaris Inc., New York, NY, USA). All radiographic measurements for global sagittal parameters and adjacent disc were performed whilst positioned at a central location based upon standardized techniques, including the coronal C7 plumbline for the sagittal vertical axis (SVA) (offset of the C7 plumbline relative to S1), pelvic tilt (PT), pelvic incidence (PI), LL (lordotic angle from the superior endplate of L1 to the superior endplate of S1), PI mines LL (PI-LL), sacral slope (SS), and thoracic kyphosis (TK) [7]. The adjacent segment was defined as being the superior level of the index fusion level. The morphological changes in adjacent segments were measured through (1) index lordosis (IL): “the angle subtended by the superior endplate line and the inferior endplate line of segments with an interbody cage. However, the IL at L5-S1 was measured as the angle subtended by the superior endplate line of L5 and the superior endplate line of S1” [8]; and (2) segmental lordosis (SL): “the angle subtended by the superior endplate line of the segment superior to the fusion level and the inferior endplate line of the segment with an interbody cage. However, the inferior line of SL at the L5-S1 level was measured as the angle subtended by the superior endplate line of S1”; (3) anterior disc height (ADH): “measured in the planes of the anterior surfaces of the adjacent vertebral bodies, where the distances between the adjacent superior and inferior endplates were the shortest” [9] of the adjacent segment; (4) posterior disc height (PDH): “measured in the planes of the posterior surfaces of the adjacent vertebral bodies, where the distances between the adjacent superior and inferior endplates were the shortest” [9] of the adjacent segment; and (5) adjacent segment disc angle (ASDA): defined as the angle subtended by the superior line and inferior line of the vertebral disc of the adjacent segment (Figure 1). The degeneration status of the adjacent discs was evaluated via pre-OP sagittal T2-weighted magnetic resonance imaging (MRI) in accordance with the modified Pfirrmann grading system by Griffith et al. [10,11]. The modified system comprises 8 grades for lumbar disc degeneration providing better discrimination of baseline degeneration severity of the adjacent discs. Grade 1 to 5 demonstrated hyper- to hypointensity of the signal from the nucleus and inner fibers of the anulus with normal disc height. Grade 6 to 8 were defined as a <30%, 30–60%, >60% reduction in disc height, respectively, compared to Grade 5.

### 2.4. Outcome Measurements

Standardized self-reported HRQOLs consisted of: (1) European Quality of life in 5-dimensional scale (EQ-5D) [12]; (2) Visual Analog Scale of Pain (VASP) measured in total (VASP-Total), in Back (VASP-Back); and (3) the Oswestry Disability Index (ODI) [13] and were obtained at pre-OP baseline, post-OP, and 2-year follow-up. The ODI is a validated disease specific instrument for assessment of spinal disorders consisting of a 10-item ordinal scale with 6 response alternatives for each item. The total score ranges from 0 to 100, where 100 indicates the worst possible disability. Pain is assessed using a pain index which is the mean of VAS-scores for ‘‘pain right now’’ and ‘‘worse pain last week’’.

**Figure 2:**
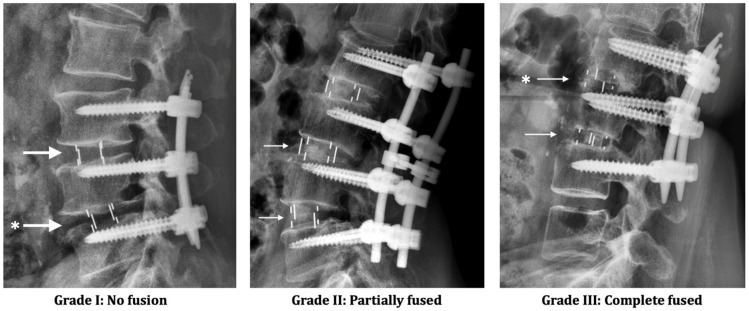
Grading for fusion status. Arrow (→), interbody cage in position. Asterisk (*), the subsidence of LIF cage.

The definitions of radiological ASD are defined as described by Nakashima et al. [14] from plain X-rays: (1) narrowing of disc height by >3 mm; (2) posterior opening >5°; and (3) progress of slippage >3 mm in comparison with pre-OP sagittal radiographs. K.-K.T., and F.-W.H. reviewed each of the flexion–extension plain films (unblinded) and recorded the grading for the fusion status. Each fusion level was evaluated separately by the Hutter method [15] according to the Santos criteria [16,17] of fusion grading at 2-year follow-up: (1) Grade I: No fusion. Any motion or radiolucency around the device; (2) Grade II: Partially fused. No motion around the device without definite bony opacity formation in/around the cage; and (3) Grade III: Complete fused: No motion or radiolucency around the device with definite bony opacity formation in/around the cage (Figure 2). Fusion rate was calculated by the proportion of grade II and grade III amongst the study cohort.

### 2.5. Statistical Analyses

Demographic factors and clinical data, including age, body mass index (BMI), gender, surgical technique, and length of post-OP hospital stays were all recorded. Normality of data was determined via the Shapiro–Wilk test. Continuous variables were described through mean and standard deviation. Changes in parameters were assessed via a paired *t*-test. Comparison analyses were conducted via either one-way analysis of variance (ANOVA), the Kruskall–Wallis test or Mann–Whitney test according to the appropriate models. Statistical analyses were 2-sided and *p* < 0.05 was considered statistically significant. All statistical analyses were performed using SPSS version 24 (IBM, Armonk, New York, NY, USA).

## 3. Results

### 3.1. Patient Population Demographics

After matching forage and gender, we enrolled a total of 50 patients in this study, including 25 who received ALIF and 25 who received OLIF with posterior instrumentation spinal fusion (Table 1). Amongst these patients, the mean age was 56.6 ± 5.0 years with 30 (60%) of them being female. The mean BMI was 25.1 ± 3.8, and the mean hospital stay was 7.0 ± 2.0 days. Additionally, 38 (76%) patients were diagnosed with spondylolisthesis for surgical indication, whilst 12 (24%) patients were diagnosed with spondylosis. Most patients received a single level fusion (*n* = 41, 82%) whereas L4/L5 (*n* = 24, 48%) was the most prevalent index correction level. The fusion rate of all patients was 100%, with an 84% complete fused rate. In accordance with the modified Pfirrmann grading system [10], 28% of discs were graded as Grade 3 whilst 44% were graded as Grade 4. There were no differences in fusion status and pre-OP adjacent degeneration status between ALIF and OLIF. No patients received revision surgery or re-operation for adjacent discs during the follow-up period.

### 3.2. Morphologic and Functional Outcomes of All Patients

**Figure 3:**
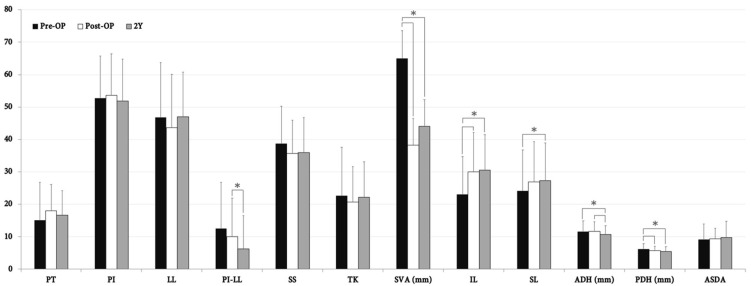
Radiographic outcome measured at pre-OP, post-OP, and 2-year follow-up amongst all patients (*n* = 50). *, statistically significant between measurements (*p* < 0.05). PT, pelvic tilt; PI, pelvic incidence; LL, lumbar lordosis; PI-LL, PI minus LL; SS, sacral slope; TK, thoracic kyphosis; SVA, sagittal vertical axis; IL, index lordosis; SL, segmental lordosis; ADH, anterior disc height; PDH, posterior disc height; ASDA, adjacent segment disc angle; Y, year.

The change in adjacent disc morphology and sagittal parameters of all patients is demonstrated in Figure 3. At post-OP follow-up, IL demonstrated a significant increase whilst PDH had decreased. At 2-year follow-up, both IL and SL had significantly increased whilst ADH and PDH had opposingly decreased compared to the pre-OP baseline. Additionally, ADH had significantly decreased during the 2-year follow-up period. When considering global sagittal parameters, SVA had significantly improved after the operation and maintained adequate correction as well at 2-year follow-up. PI-LL mismatch was significantly corrected only during the follow-up period. There were no statistically significant differences between ASDA, PT, PI, LL, SS, and TK during the 2-year follow-up period amongst these patients.

### 3.3. Morphologic and Functional Outcomes in the ALIF and OLIF Groups

**Figure 4:**
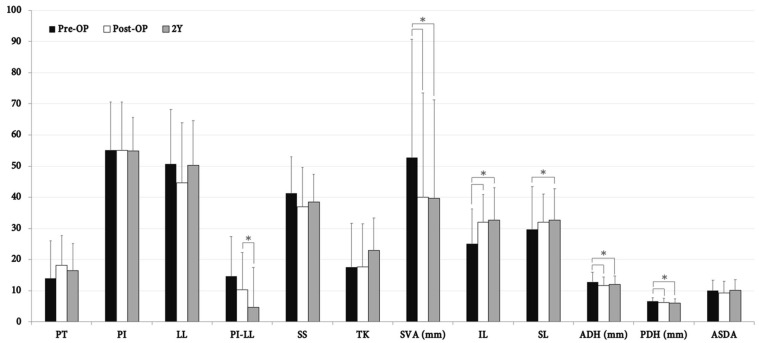
Radiographic outcome measured at pre-OP, post-OP, and 2-year follow-up amongst ALIF group (*n* = 25). *, statistically significant between measurements (*p* < 0.05). PT, pelvic tilt; PI, pelvic incidence; LL, lumbar lordosis; PI-LL, PI minus LL; SS, sacral slope; TK, thoracic kyphosis; SVA, sagittal vertical axis; IL, index lordosis; SL, segmental lordosis; ADH, anterior disc height; PDH, posterior disc height; ASDA, adjacent segment disc angle; Y, year.

**Figure 5:**
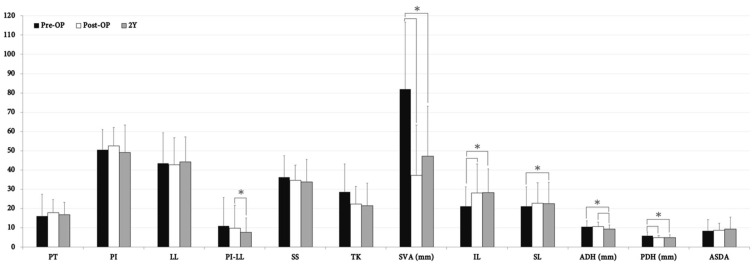
Radiographic outcome measured at pre-OP, post-OP, and 2-year follow-up amongst OLIF group (*n* = 25). *, statistically significant between measurements (*p* < 0.05). PT, pelvic tilt; PI, pelvic incidence; LL, lumbar lordosis; PI-LL, PI minus LL; SS, sacral slope; TK, thoracic kyphosis; SVA, sagittal vertical axis; IL, index lordosis; SL, segmental lordosis; ADH, anterior disc height; PDH, posterior disc height; ASDA, adjacent segment disc angle; Y, year.

Consistent with the outcome of all patients, IL demonstrated a significant increase whilst PDH decreased after surgery at the earliest post-OP follow-up in both the ALIF and OLIF groups. IL and SL had significantly increased, whilst ADH and PDH had opposingly decreased at the 2-year follow-up period when compared to the pre-OP baseline. In terms of adjacent disc height, the only difference between the two groups was the timing of the morphological change in ADH. The ADH decreased at the earliest post-OP follow-up with no further differences until the 2-year end time in the ALIF group. By contrast, ADH only significantly dropped at the 2-year post-OP follow-up in the OLIF group (Figure 4 and Figure 5).

After matching for gender and age, patients receiving ALIF and OLIF were compared for radiographic (Table 2) and functional outcomes (Table 3). All baseline radiographic parameters demonstrated no significant differences. IL, SL and PDH were smaller at post-OP and 2-year follow-up, whilst ADH was smaller only at 2-year follow-up in the OLIF group. ASDA and sagittal parameters, including PT, PI, LL, PI-LL, SS, TK, and SVA, demonstrated no significant differences between the two groups (Table 2).

The comparison of functional outcomes between ALIF and OLIF is presented in Table 3. No statistical difference was identified in EQ-5D, ODI, VASP-Total and VASP-Back between LIFs at the pre-OP, post-OP and 2-year follow-up time points.

## 4. Discussion

In this study, we reported the morphological change in adjacent disc, sagittal alignment, and functional outcomes. Compared to the pre-OP baseline set 2 years previously, it was discovered that IL and SL were greatly restored, whilst PDH decreased. In terms of spinal sagittal parameters, SVA and PI-LL had been corrected after the operation. The comparison between LIFs showed that IL, SL and PDH were smaller at post-OP and 2-year follow-up. On the other hand, ADH was smaller only at 2-year follow-up in the OLIF group, and this indicates a flatter disc and lumbar spine morphology. However, there was no difference in fusion status, pre-OP adjacent disc degeneration status and functional outcomes between LIFs. It is common to read studies about patients suffering from ASD after receiving posterior lumbar spine arthrodesis and posterior LIFs. In comparison, this is the first report regarding morphological change in the adjacent segment after patients have received anterolateral LIFs, which includes ALIF and OLIF, as well as involving similar fusion status, sagittal alignments, and patients’ self-reported outcomes. At the same time, studies comparing the incidence rate of ASD of ALIF and OLIF after surgery are scarce [18].

The radiographic outcomes of adjacent discs in LIFs were well described recently [1,2,3,4,5,6,8,19,20,21]. Aging factors, adjacent segment decompression, fusion length, fusion technique, intraoperative superior facet joint violation, sagittal balance, and pre-OP pathologies may all be related to adjacent disc shape after lumbar fusions [22,23]. It has been proposed that surgical factors and outcomes may play an important role in predicting and preventing ASD amongst patients receiving LIFs [23]. Several spinal radiographic parameters have also been considered as predictive factors for ASD amongst spinal fusion patients. A high degree of kyphosis results in a greater incidence of radiographically confirmed ASD after cervical arthrodesis [24,25,26]. Studies have addressed the importance of PI in predicting the occurrence of ASD in spinal disease patients [14,24]. Moreover, patients with a pre-OP PT >22.5° may suffer a 5.1 times greater risk of developing ASD after receiving transforaminal LIF (TLIF) [27]. In this study, aging factors, fusion length, and proceeded cautious intraoperative management were controlled to minimize the predisposition of the confounding factors that lead to ASD.

The restoration of lordosis is considered as the surgical goal in LIF surgery since loss of lordosis results in poor outcomes. A human cadaveric spine study showed hypolordotic fusion increased the flexion–extension motion in the adjacent disc [28]. Other evidence supports the mechanism by reporting that the hypolordotic fusion of the index correction level results in compensating hyperlordosis, which leads to significant degenerative change in the adjacent disc [5]. In a study comparing ALIF, lateral LIF (LLIF), and posterior LIF (PLIF) for preventing the development of ASD in L4/5 spondylolisthesis [18], the authors found the ALIF group were associated with lower ASD incidence rate and better outcomes in terms of LL compared to PLIF. They suggested that ALIF produced better sagittal alignment than the other fusion methods, whilst on the other hand, the lack of anterior longitudinal ligament (ALL) resection seemed to be the major challenge in the creation of a large post-OP lordosis in LLIF and PLIF. Moreover, the facetectomies and long muscle retraction time in PLIF may cause an increase in the rate of ASD, which also demonstrates the benefits of anterolateral approaches.

In line with previous studies, the immediate change in IL and PDH in the current study described a wedge-shape conformation of adjacent disc after surgery, demonstrating a rapid alternation of pressure distribution whilst restoring the LL through implantation of interbody cages. The decrease in ADH and PDH indicated a flatten adjacent disc at 2-year follow-up, which may result from compression of the anterior and middle column of the spine after anterolateral LIFs. However, the ALIF and OLIF techniques have each proven their efficacy for the restoration of the sagittal parameters after surgery and could be sustained for 2 years in both fusion groups, resulting in satisfactory functional outcomes and adjacent disc quality, which was in line with previous reports [18,29], especially in IL, SL, PI-LL and SVA. We also found an improvement in reginal lordosis of adjacent segments via the significant increase in SL whilst ASDA remained simultaneously unchanged at 2-year follow-up. Throughout the study, there were no radiological ASD diagnosed or revision surgery for ASD during the follow-up period. Compared to the posterior approach, anterolateral access allows ALIF and OLIF to facilitate a more aggressive correction of lordosis and foraminal height restoration by maximizing the implant size and fusion surface area. As a result, we have purposed that restoration of lordosis and the sagittal axis in a treated spinal segment after ALIF and OLIF may be able to minimize the acceleration of ASD. Moreover, ALIF and OLIF have been reported as a safe and efficient method to treat ASD after posterior lumbar fusion [30,31], with shorter operative time, hospital stay, lesser blood loss, and lower risk of dural injury compared to posterior surgeries for degenerative spine disease.

When comparing LIFs in clinical practice, both ALIF and OLIF are able to obliviate posterior column destruction and provide surgeons with a wider space for bony graft placement. In the current study, the ALIF group showed greater IL, SL, ADH, and PDH at 2-year follow-up when compared to the OLIF group. Regarding interbody placement, ALIF allows for a cage with a greater lordotic angle, since the ALL is released intraoperatively [32]. However, when approaching segments above L4 in ALIF, vessel injuries could be the greatest concern [18]. At L5/S1, there no significant difference in LL and patient-reported outcomes, whether choosing either ALIF or OLIF for correction [33]. Despite studies claiming the potential of OLIF at L5/S1 level, with promising outcomes, especially in obese patients when ALIF bears higher risk, we preferred to choose ALIF when encountering L5/S1 surgery due to its better visualization and the easier approach at this level [34,35]. The minimally invasive approach allows for a shorter recovery time, and corridor neuromonitoring is not required when compared with LLIF. However, patients experiencing central canal stenosis or high-grade spondylolisthesis were relative contraindicated for OLIF. As our study stated, both ALIF and OLIF offered positive clinical results and adequate IL restoration; therefore, patient selection through appropriate surgical indications and correction levels is vital. Finally, promising results have been reported in treating ASD after posterior lumbar fusion with ALIF and OLIF [30,36]. However, the relationship and mechanism between prevention and treatment of ASD via ALIF and OLIF remain undetermined.

As with most studies, the design of the current study is subject to limitations. First, we only recruited 50 patients, which may be too small a population to establish statistical power and draw robust conclusions. Nevertheless, we matched the patients to strengthen the statistical power level, with the attendings who performed the surgeries all trained in the same medical center whilst surgical tools and implants were restrained unitarily, which could minimize any inconsistencies. Secondly, we tried to determine the efficacy of ALIF and OLIF by assessing the alignment restoration and the occurrence of post-OP ASD. However, there were numerous factors to assess the progression of ASD, such as degeneration progression of adjacent discs, adjacent posterior zygapophyseal joints, the relationship between pedicle screw and superior endplate [37] that were not evaluated. A proper subgroup analysis according to the degeneration grade and its progression over time should be designed in any future study. Finally, while ALIF and OLIF have different surgical indications and contraindications, selection bias could not be neglected. To be precise, other than degenerative disc disease, sagittal or coronal deformity correction were also considered to be an indication for OLIF [38]. The synergistic effect in sagittal correction via OLIF may amplify the angle observed and over-rate the lordosis restoration [39]. The intrinsically greater lordotic curvature in more distal segments could be the confounding factor when comparing the angle corrected between the two groups [40]. However, the segments treated in the two groups disclosed no significant differences in our study, and therefore the concern may be minimal. In short, we regard the results of our study to be trustworthy but feel that more research and cases are still required to establish an even more thorough evaluation regarding the use of both ALIF and OLIF.

## 5. Conclusions

In our study, although the adjacent disc shape may be flattened at 2-year follow-up after receiving ALIF and OLIF, the improvement in LL, IL, SL, and sagittal alignments showed promising results in ASD avoidance and good patient-reported outcomes. Although the ALIF group showed greater improvements in IL and SL, the ALIF and OLIF procedure showed no differences in fusion status, functional outcomes, ASD incidence rate, and sagittal parameters. In this case, patient selection remains crucial when deciding between the two methods.

## Figures and Tables

**Table 1:** Demographic data of the study population stratified by lumbar interbody fusion types.

KERRYPNX	ALIF	OLIF	Overall	*p*-Value
Patient number	25	25	50	
Total correction levels	30	27	57	0.85
Age (years)	57.2 ± 3.7	58.1 ± 4.2	56.6 ± 5.0	0.63
BMI (Kg/m^2^)	24.9 ± 2.9	25.9 ± 3.0	25.1 ± 3.8	0.82
Hospital stays (days)	7.0 ± 1.5	7.0 ± 2.5	7.0 ± 2.0	0.90
Gender				1
Male	10 (40%)	10 (40%)	20 (40%)	
Female	15 (60%)	15 (60%)	30 (60%)	
Pre-OP diagnosis				0.87
Spondylolisthesis	18 (72%)	20 (80%)	38 (76%)	
Spondylosis	7 (28%)	5 (20%)	12 (24%)	
Fusion levels				0.88
1	20 (80%)	21 (84%)	41 (82%)	
2	5 (20%)	3 (12%)	8 (16%)	
3	0 (0%)	1 (4%)	1 (2%)	
Index fusion level				0.09
L2/L3	0 (0%)	1 (3.7%)	1 (1.8%)	
L3/L4	4 (13.3%)	11 (40.7%)	15 (26.3%)	
L4/L5	9 (30%)	15 (55.6%)	24 (42.1%)	
L5/S1	17 (56.7%)	0 (0%)	17 (29.8%)	
Fusion status				0.72
Grade I	0 (0%)	0 (0%)	0 (0%)	
Grade II	4 (13%)	5 (19%)	9 (16%)	
Grade III	26 (87%)	22 (71%)	48 (84%)	
Pre-OP adjacent disc degeneration status				
Grade 1	1 (4%)	0 (0%)	1 (2%)	0.68
Grade 2	2 (8%)	3 (12%)	5 (10%)	
Grade 3	7 (28%)	7 (28%)	14 (28%)	
Grade 4	12 (48%)	10 (40%)	22 (44%)	
Grade 5	3 (12%)	5 (20%)	8 (16%)	

Continuous data are expressed as mean ± SD. Categorical data were expressed as numbers and percentage.

**Table 2:** Comparison of radiographic outcome of the study population stratified by lumbar interbody fusion types.

	ALIF	OLIF	Overall	*p*-Value
PT (°)				
Pre-OP	13.9 ± 12.1	16.1 ± 11.3	15.1 ± 11.6	0.69
Post-OP	18.1 ± 9.6	17.9 ± 6.7	18 ± 8.1	0.98
2Y	16.4 ± 8.7	16.9 ± 6.4	16.7 ± 7.5	0.91
PI (°)				
Pre-OP	55.2 ± 15.3	50.6 ± 10.5	52.8 ± 13	0.31
Post-OP	55 ± 15.6	52.5 ± 9.6	53.7 ± 12.7	0.99
2Y	54.9 ± 10.7	49.1 ± 14.3	51.8 ± 13	0.13
LL (°)				
Pre-OP	50.6 ± 17.6	43.5 ± 15.8	46.8 ± 16.9	0.31
Post-OP	44.7 ± 19.2	42.7 ± 14.1	43.6 ± 16.5	0.84
2Y	50.2 ± 14.4	44.2 ± 12.9	47 ± 13.8	0.16
PI-LL (°)				
Pre-OP	14.6 ± 12.8	10.9 ± 14.8	12.6 ± 14.1	0.11
Post-OP	10.3 ± 12	9.8 ± 12	10 ± 11.9	0.78
2Y	4.6 ± 12.8	7.6 ± 7.5	6.2 ± 10.3	0.26
SS (°)				
Pre-OP	41.3 ± 11.7	36.3 ± 11.1	38.7 ± 11.6	0.3
Post-OP	36.9 ± 12.6	34.6 ± 7.9	35.7 ± 10.3	0.67
2Y	38.5 ± 8.9	33.8 ± 11.8	36 ± 10.7	0.07
TK (°)				
Pre-OP	17.6 ± 14.1	28.7 ± 14.4	22.6 ± 15	0.16
Post-OP	17.6 ± 13.9	22.4 ± 9	20.7 ± 11	0.45
2Y	23 ± 10.3	21.6 ± 11.5	22.2 ± 10.9	1
SVA (mm)				
Pre-OP	52.8 ± 37.9	81.9 ± 34.7	65.1 ± 38.6	0.12
Post-OP	40 ± 33.5	37.2 ± 26.2	38.2 ± 28.3	0.84
2Y	39.6 ± 31.6	47.3 ± 25.8	44 ± 28.3	0.37
IL (°)				
Pre-OP	25.1 ± 11.2	21.1 ± 10.2	23.1 ± 10.6	0.09
Post-OP	31.9 ± 8.9	28.0 ± 15.1	30.0 ± 12.2	0.03 *
2Y	32.6 ± 10.4	28.4 ± 12.2	30.5 ± 11	<0.01 **
SL (°)				
Pre-OP	29.7 ± 13.7	21.1 ± 10.2	25.1 ± 12.6	0.08
Post-OP	31.9 ± 9.2	22.8 ± 10.5	27 ± 10.8	0.01 *
2Y	32.7 ± 10.1	22.6 ± 11	27.3 ± 11.6	<0.01 **
ADH (mm)				
Pre-OP	12.8 ± 3.1	10.6 ± 3.1	11.6 ± 3.3	0.07
Post-OP	11.7 ± 2.7	10.7 ± 2.4	11.2 ± 2.6	0.29
2Y	12 ± 2.7	9.5 ± 2.1	10.7 ± 2.7	<0.01 **
PDH (mm)				
Pre-OP	6.6 ± 1.2	5.8 ± 2.1	6.2 ± 1.8	0.05
Post-OP	6.2 ± 1.3	4.9 ± 1.1	5.5 ± 1.4	<0.01 **
2Y	6.1 ± 1.3	5 ± 1.4	5.5 ± 1.5	<0.01 **
ASDA (°)				
Pre-OP	10 ± 3.3	8.5 ± 5.8	9.2 ± 4.8	0.05
Post-OP	9.3 ± 3.7	8.8 ± 3.6	9 ± 3.6	0.52
2Y	10.1 ± 3.4	9.4 ± 6.2	9.7 ± 5.1	0.12

Continuous data are expressed as mean ± SD. * *p* < 0.05, ** *p* < 0.01: consider statistically significant between ALIF and OLIF. PT, pelvic tilt; PI, pelvic incidence; LL, lumbar lordosis; PI-LL, PI minus LL; SS, sacral slope; TK, thoracic kyphosis; SVA, sagittal vertical axis; IL, index lordosis; SL, segmental lordosis; ADH, anterior disc height; PDH, posterior disc height; ASDA, adjacent segment disc angle; Y, year.

**Table 3:** Comparison of functional outcome of the study population stratified by lumbar interbody fusion types.

	ALIF	OLIF	Overall	*p*-Value
EQ-5D				
Pre-OP	11.3 ± 1.6	11.6 ± 0.7	11.5 ± 1.2	0.49
Post-OP	6.6 ± 2.2	7.3 ± 1.9	7 ± 2.1	0.25
2Y	7.5 ± 2.1	7.1 ± 2.8	7.4 ± 2.4	0.37
ODI				
Pre-OP	49.3 ± 11.9	49.4 ± 11.5	49.4 ± 11.5	0.98
Post-OP	20.2 ± 16.8	27.4 ± 15.4	23.7 ± 16.3	0.15
2Y	27.6 ± 17.4	29.1 ± 18.7	28.2 ± 17.9	0.69
VASP-Total				
Pre-OP	8.2 ± 1.5	10.3 ± 11.8	9.4 ± 8.7	0.43
Post-OP	2.3 ± 2.2	4.1 ± 6.3	3.2 ± 4.8	0.22
2Y	3.3 ± 2.1	2.8 ± 1.6	3.1 ± 1.9	0.25
VASP-Back				
Pre-OP	6 ± 3.2	7.3 ± 2.4	6.72 ± 2.8	0.1
Post-OP	1.6 ± 1.7	2.4 ± 1.9	2 ± 1.8	0.15
2Y	2.3 ± 2.05	2.2 ± 2.13	2.3 ± 2.07	0.81

Continuous data are expressed as mean ± SD. PT, pelvic tilt; PI, pelvic incidence; LL, lumbar lordosis; PI-LL, PI minus LL; SS, sacral slope; TK, thoracic kyphosis; SVA, sagittal vertical axis; IL, index lordosis; SL, segmental lordosis; ADH, anterior disc height; PDH, posterior disc height; ASDA, adjacent segment disc angle; Y, year.

## Data Availability

All data are available upon reasonable request from the corresponding author.
